# Kerbs von Lungren 6 antigen is a marker of alveolar inflammation but not of infection in patients with acute respiratory distress syndrome

**DOI:** 10.1186/cc6785

**Published:** 2008-01-23

**Authors:** Nazim Nathani, Gavin D Perkins, William Tunnicliffe, Nick Murphy, Mav Manji, David R Thickett

**Affiliations:** 1 Lung Injury and Fibrosis Treatment Program, Department of Medical Sciences, The Medical School, University of Birmingham, Edgbaston, Birmingham B15 2TT, UK; 2 Department of Intensive Care Medicine, Birmingham Heartlands Hospital, Birmingham B9 5SS, UK; 3 Department of Critical Care, University Hospital Birmingham, Birmingham, B152TH, UK; 4 Lung Injury and Fibrosis Treatment Program, Nuffield House, Queen Elizabeth Hospital, Department of Medicine, University of Birmingham B15 2TH, UK

## Abstract

**Background:**

Kerbs von Lungren 6 antigen (KL-6) is expressed on the surface of alveolar type II cells, and elevated plasma and epithelial lining fluid levels of KL-6 have previously been shown to correlate with the severity of disease and survival in acute respiratory distress syndrome (ARDS). The relationship between alveolar inflammation and KL-6 measurements has not been ascertained. We hypothesized that the elevation of KL-6 in ARDS is dependent upon the severity of neutrophilic inflammation. Furthermore we were interested in the relationship between significant alveolar infection and KL-6 levels.

**Methods:**

Plasma arterial samples were collected from ARDS patients on day 1 and when possible on day 4 along with bronchoalveolar lavage fluid (BALF) samples on the same day. Bacterial growth in the BALF was determined by quantitative cultures and was defined as significant at counts >1 × 10^4 ^colony-forming units.

**Results:**

Plasma KL-6 levels in ARDS patients were elevated compared with at-risk control individuals (*P *= 0.014) and with normal control individuals (*P *= 0.02). The plasma KL-6 level correlated with the Murray Lung Injury Score (*r *= 0.68, *P *= 0.001) and with BALF KL-6 (*r *= 0.3260, *P *= 0.04). The BALF KL-6 level was detectable in all ARDS cases and was lower on both day 0 and day 4 in those who survived. BALF KL-6 also correlated with the BALF myeloperoxidase activity (*r *= 0.363, *P *= 0.027), with the BALF cell count per millilitre (*r *= 0.318, *P *= 0.038), with BALF epithelial-cell-derived neutrophil attractant 78; (*r *= 0.37, *P *= 0.016) and with BALF vascular endothelial growth factor (*r *= 0.35, *P *= 0.024). The BALF KL-6 level of ARDS patients with significant pathogenic bacterial growth was similar compared with those without significant infection.

**Conclusion:**

KL-6 may represent a useful marker of alveolar type II cell dysfunction in ARDS since the levels reflect the severity of lung injury and neutrophilic inflammation. KL-6 release across the alveolar epithelial barrier is associated with a poor prognosis. The pathophysiological roles of KL-6 in the development of ARDS warrant further study.

## Introduction

Acute respiratory distress syndrome (ARDS) is characterized by disruption of the alveolar–capillary barrier and by neutrophilic inflammation [1,2]. The pathology of ARDS consists of inflammation in the alveolar space, with a predominance of neutrophils. Analysis of bronchoalveolar lavage fluid (BALF) from patients with ARDS has shown increased numbers of activated neutrophils in the early stages of ARDS [3]. The number of neutrophils in BALF relates to the severity of lung injury [4], and the persistence of neutrophils is associated with increased mortality [5].

Kerbs von Lungren 6 antigen (KL-6) is a high-molecular-weight glycoprotein, classified as cluster 9 (MUC1) of lung tumour and differentiation antigens according to the findings of immunohistochemical and flow cytometry studies [6]. KL-6 splits off at the S–S bond near the epithelial membrane surface and becomes distributed in pulmonary epithelial lining fluid. This glycoprotein is predominantly expressed on alveolar type II cells in the lung, with expression increasing in proliferating, regenerating or injured type II cells more than normal type II cells [7-9].

Serum levels of KL-6 are elevated in a variety of interstitial lung diseases that are characterized by alveolar epithelial cell damage. Serum KL-6 concentrations are associated with alveolar–epithelial barrier dysfunction as they have been shown to correlate with indices of alveolar–capillary permeability [10].

Two studies have examined the serum and oedema fluid KL-6 concentrations in adult patients with acute lung injury (ALI); both studies found elevated plasma levels [8,11]. The study by Ishizaka and colleagues demonstrated elevated levels of KL-6 in oedema fluid collected by bronchoscopic microsampling of patients with ARDS, and confirmed primary human epithelial cell production of KL-6 in response to proinflammatory cytokines [8]. Both studies suggest that measuring KL-6 could be a valuable marker of poor prognosis in clinical ALI, although several questions regarding the relevance of elevated KL-6 in ALI remain. Are KL-6 concentrations in BALF prognostic in ALI? Does the degree of alveolar inflammation correlate with levels of KL-6 within the lung? Does active alveolar infection influence the KL-6 levels in ARDS? Can KL-6 be used to stratify risk for ARDS in patient groups or individuals?

The present study demonstrates for the first time that the BALF KL-6 concentration is elevated in patients with ARDS. BALF KL-6 correlated with plasma KL-6 levels and was related to the severity of neutrophilic inflammation. Alveolar infection does not seem to determine the levels of KL-6 in either BALF or plasma. We suggest that KL-6 may represent a useful marker of alveolar type II cell dysfunction in ARDS since the levels reflect the severity of lung injury and neutrophilic inflammation but not the presence of alveolar infection.

## Materials and methods

### Participants

Consecutive patients with ARDS were studied within 48 hours of admission to the critical care unit of University Hospital Birmingham (Birmingham, UK) between 2004 and 2006. The nature of illness precluded obtaining prospective consent in those with ARDS; in this group, whenever possible, retrospective written informed consent was obtained. All other study participants gave written informed consent. The study was approved by the local research ethics committee.

Thirty patients were identified during the study as having ALI or ARDS according to the American–European consensus statement [12]. Patients were ventilated using pressure-controlled ventilation aiming for tidal volumes of 6 ml/kg. Bronchoscopy and bronchoalveolar lavage (was performed in all patients immediately following inclusion and, when possible, 4 days later (*n *= 15). Of the 15 patients in whom repeat bronchoscopy could not be performed, eight died, three were extubated, and four had contraindications to bronchoscopy.

The patients' demographic characteristics were recorded at baseline. The Acute Physiology and Chronic Health Evaluation II score, the Simplified Acute Physiology Score II and the predicted intensive care unit mortality were recorded as global markers of disease severity. The Murray Lung Injury Score and the Sequential Organ Failure Assessment score were recorded daily.

Exclusion criteria were age <18 years, severe obstructive airways disease, neutropenia (defined as neutrophil count <0.3 × 10^9 ^l), and/or known/suspected brain stem death.

Healthy volunteers (*n *= 10, mean age 49 years, nonsmoking, free from respiratory disease) were defined as normal individuals. Twelve ventilated patients with risk factors for ALI but who had not developed ALI at the time of recruitment were included as 'at-risk' intensive therapy unit control individuals (see Table 1).

**Table 1:** 

Parameter	ARDS patients	Individuals at risk of ARDS	*P *value
Number of patients	30	12	-
Male:female (*n*)	17:13	7:5	-
Mean age (years)	62 (3.5)	59 (4.5)	0.6
Lung Injury Score	2.7 (0.42)	1.2 (0.3)	0.001
PaO_2_:FiO_2 _ratio (kPa)	16.1 (7)	33.5 (12.1)	0.0001
Acute Physiology and Chronic Health Evaluation II score	28.9 (5.7)	21.7 (8.9)	0.139
Simplified Acute Physiology Score II	55.8 (16.62)	54.8 (12.8)	0.639
Sequential Organ Failure Assessment score	14.9 (3.3)	7.1 (4.3)	0.001

### Bronchoscopy

The bronchoscope was wedged into to a subsegmental bronchus in the middle lobe and 150 ml of 0.9% saline was instilled in three 50 ml aliquots [3]. The BALF was aspirated and placed immediately on ice until processing. Fifteen millilitres of blood was collected simultaneously into lithium heparin tubes (Becton Dickinson, Birmingham, UK) and was stored on ice until processing.

### Sample processing

The BALF volume was measured and then filtered through a single layer of surgical gauze to remove debris, and was centrifuged at 500 × *g *for 5 minutes. The supernatant was removed and stored at -80°C until subsequent analysis. Whole blood was spun at 500 × *g *for 10 minutes, and the plasma was removed and stored at -80°C until analysis.

### ELISA and myeloperoxidase assay measurements

KL-6 was measured by ELISA (Eisai Corporation, Tokyo, Japan) according to the manufacturer's instructions in BALF and in plasma. The intra-assay coefficient of variation was 5.1% and the inter-assay coefficient of variation was 8.9% in both BALF and plasma. BALF IL-8, epithelial-cell-derived neutrophil attractant 78 (ENA-78) and vascular endothelial growth factor (VEGF) were measured by ELISA (R&D Systems, Abingdon, UK) according to the manufacturer's instructions. Myeloperoxidase activity was measured by chromogenic substrate assay as previously described (expressed as units of activity per millilitre) [13].

### Bronchoalveolar lavage fluid microbiology culture

The BALF from patients with ARDS was quantitatively cultured. A growth of a pathogen >10^4 ^colony-forming units/ml was considered to represent significant active infection.

### Statistical methods

The Ryan–Joiner normality test was used to test the distribution of the data. The plasma and BALF KL-6 data were not normally distributed, and therefore are presented as the median with 95% confidence interval (CI) for the median difference. Between-group comparisons were performed using the Mann–Whitney U test, and multiple group comparisons using the Kruskal–Wallis test. Correlations were made using Pearson's test on log-transformed data and are 'day 0' comparisons unless otherwise stated.

The study was considered hypothesis-generating; a power calculation was therefore not performed. All statistics were performed using Minitab 14. *P *< 0.05 was considered statistically significant.

## Results

### KL-6 is elevated in the plasma of patients with ARDS and reflects outcome

**Figure 1:**
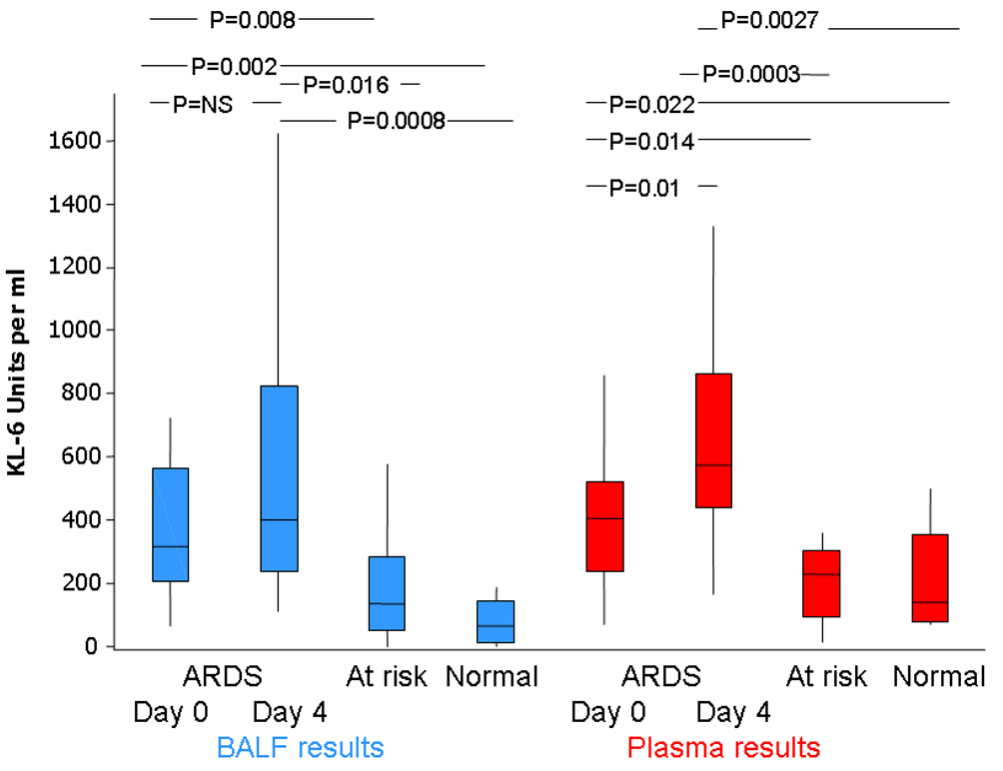
Kerbs von Lungren 6 antigen levels in plasma and in bronchoalveolar lavage fluid. Plasma and bronchoalveolar lavage fluid (BALF) levels of Kerbs von Lungren 6 antigen (KL-6) are persistently elevated in acute respiratory distress syndrome (ARDS) patients compared with normal and at-risk individuals. Plasma levels measured by ELISA.

The median plasma KL-6 level on day 0 (422 u/ml) was significantly increased compared with both normal control individuals (137.1, 95% CI median difference = 31 to 397, *P *= 0.022) and individuals at risk of ARDS (median = 222 u/ml, 95% CI median difference = 22.4 to 276.4, *P *= 0.014). Plasma levels from ARDS patients increased significantly from day 1 (422 u/ml) to day 4 (588.7 u/ml, 95% CI = 120 to 189, *P *= 0.01) (Figure 1).

**Figure 2:**
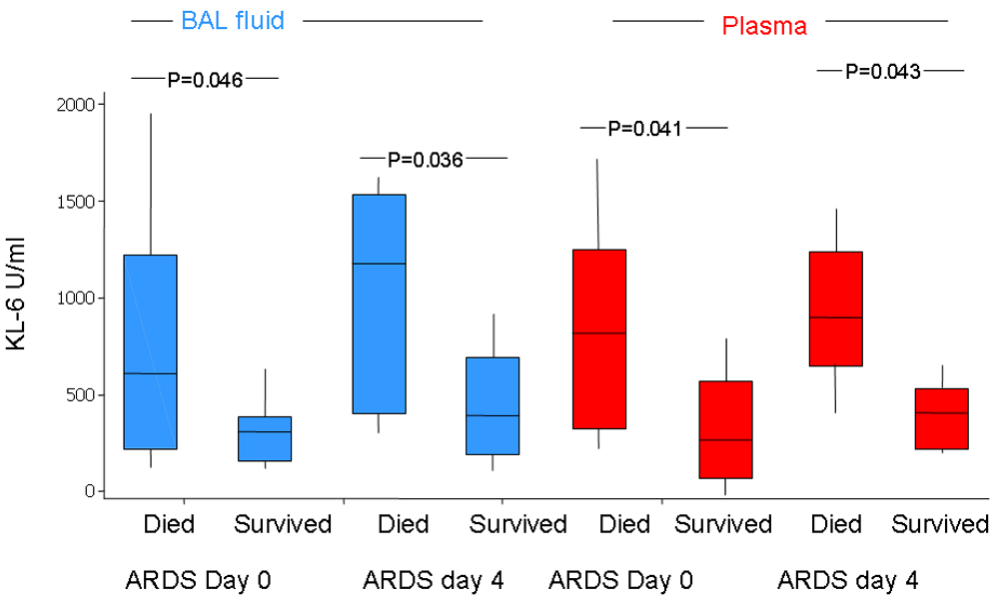
Kerbs von Lungren 6 antigen levels in survivors and nonsurvivors. Plasma and bronchoalveolar lavage fluid (BALF) Kerbs von Lungren 6 antigen (KL-6) levels are increased in acute respiratory distress syndrome (ARDS) patients who subsequently die of the disease.

**Figure 3:**
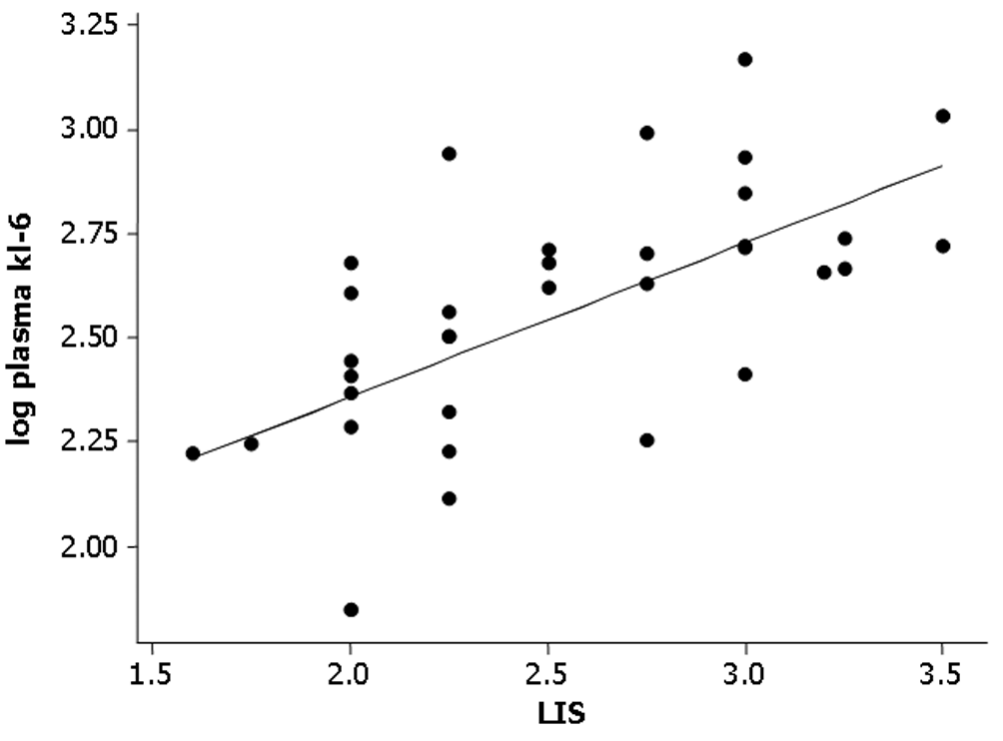
Correlation between Kerbs von Lungren 6 antigen and the Murray Lung Injury Score. Log plasma levels of Kerbs von Lungren 6 antigen (KL-6) correlate with the Murray Lung Injury Score (LIS) (*r *= 0.68, *P *= 0.001).

Patients who died had significantly higher plasma KL-6 concentration than survivors at both day 0 (median died 738 u/ml versus survived 310 u/ml, *P *= 0.041) and day 4 (median died 909.8 u/ml versus survived 414.5 u/ml, *P *= 0.043) (Figure 2). The plasma KL-6 level correlated significantly with the Murray Lung Injury Score (*r *= 0.68 *P *= 0.001) (see Figure 3) but not with the systemic injury scores – Simplified Acute Physiology Score II, Acute Physiology and Chronic Health Evaluation II or Sequential Organ Failure Assessment score (data not shown).

Both central venous blood and arterial blood were measured in 12 patients with ARDS to look for a transpulmonary gradient in KL-6, but the values were similar (median arterial 314 u/ml versus venous 312 u/ml, *P *= 0.6) (data not shown).

### BALF KL-6 level is elevated in patients with ARDS and reflects outcome

BALF KL-6 was detectable in all patients with ARDS (day 0 ARDS median BAL KL-6 concentration, 305 u/ml), and was higher than both control individuals (median = 67 u/ml, 95% CI median difference = 125 to 424, *P *= 0.002) and at-risk individuals (median = 169.4, 95% CI median difference = 35 to 380, *P *= 0.008). KL-6 remained elevated at day 4 (median = 401 u/ml) compared with both control individuals (median = 67 u/ml, 95% CI median difference = 125.7 to 424.9, *P *= 0.0008) and at-risk individuals (median = 169.4 u/ml, 95% CI median difference = 59.3 to 725.6, *P *= 0.016) (Figure 1). Plasma and BALF KL-6 levels on day 0 correlated in patients with ARDS (*r *= 0.3260, *P *= 0.04) (data not shown).

BAL KL-6 did not correlate either with the Lung Injury Score, the Simplified Acute Physiology Score II, or the Acute Physiology and Chronic Health Evaluation II score. The BALF levels of KL-6 were significantly higher in those that died compared with those who survived, both on day 0 (median survived 308 u/ml versus died 608 u/ml, *P *= 0.046) and on day 4 (median died 1,179 u/ml versus survived 360 u/ml, *P *= 0.036) (Figure 2).

### BALF KL-6 correlates with cellular inflammation and BALF chemokines and the type II epithelial cell product VEGF

**Figure 4:**
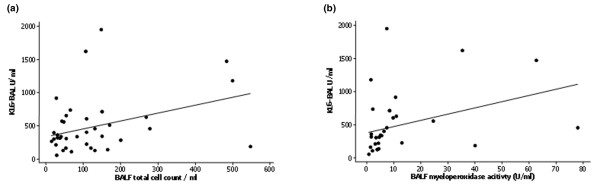
Kerbs von Lungren 6 antigen correlation with bronchoalveolar lavage fluid cell count and myeloperoxidase activity. Bronchoalveolar lavage fluid (BALF) Kerbs von Lungren 6 antigen (KL-6) levels correlate with **(a) **the BALF cell count per millilitre (*r *= 0.318, *P *= 0.038) and **(b) **the BALF myeloperoxidase activity (*r *= 0.363, *P *= 0.027) in patients with acute respiratory distress syndrome.

The BALF KL-6 concentration correlated with the BAL cell count per millilitre (*r *= 0.318, *P *= 0.038) and with the BAL myeloperoxidase activity (*r *= 0.363, *P *= 0.027) in patients with ARDS (Figure 4a,b).

**Figure 5:**
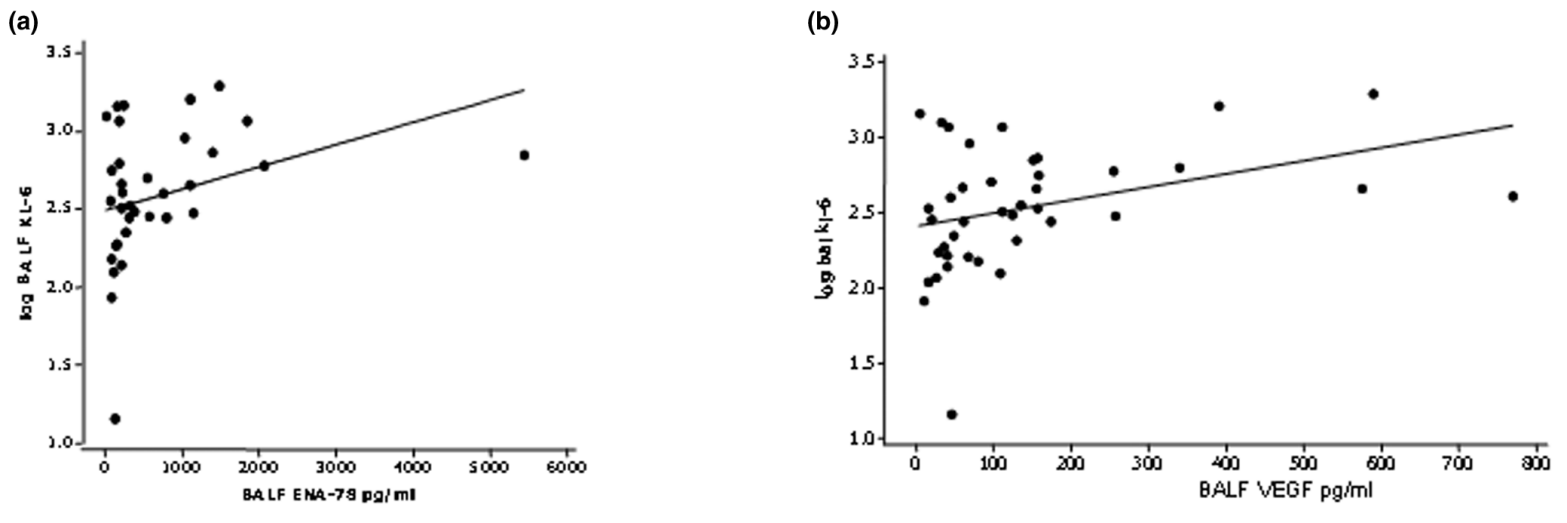
Correlation of Kerbs von Lungren 6 antigen with bronchoalveolar lavage fluid chemokine levels. Bronchoalveolar lavage fluid (BALF) Kerbs von Lungren 6 antigen (KL-6) correlates with **(a) **BALF epithelial-cell-derived neutrophil attractant 78 (ENA-78) (*r *= 0.37, *P *= 0.016) and **(b) **BALF vascular endothelial growth factor (VEGF) (*r *= 0.35, *P *= 0.024).

In addition, we evaluated the relationship between BALF KL-6 and BALF chemokine levels (IL-8 and ENA-78), which are known to be elevated in ARDS. There was no correlation between KL-6 and IL-8, but the log BALF KL-6 concentration on day 0 did correlate significantly with ENA-78 (*r *= 0.37, *P *= 0.016). In addition, there was a significant relationship between log BALF KL-6 and VEGF (*r *= 0.35, *P *= 0.024) (Figure 5a,b).

### BALF KL-6 is not influenced by the presence of active infection in ARDS patients or the mode of lung injury

Previous studies have indicated that the mode of lung injury can influence the inflammatory milieu and pulmonary mechanics of the lung in ARDS [14]. To address this indication we analysed the BALF KL-6 level by mode of lung injury and in relation to whether BALF grew significant numbers of pathogenic organisms. The mode of injury (direct versus indirect) was defined clinically according to the presumed aetiology of lung injury.

**Figure 6:**
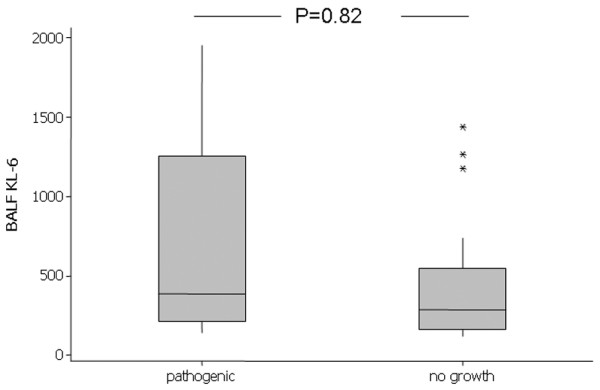
Kerbs von Lungren 6 antigen levels in patients with growth of pathogenic bacteria. Comparison of day 0 bronchoalveolar lavage fluid (BALF) Kerbs von Lungren 6 antigen (KL-6) levels in patients who had a significant growth of pathogenic bacteria (>10^4 ^colony-forming units/ml) from their lavage fluid with those showing no growth.

The presence of active alveolar infection was defined by the presence of >10^4 ^colony-forming units of pathogenic bacteria in BALF (irrespective of mode of injury). A significant growth of pathogenic bacteria was observed in 10/32 patients on day 0. The median KL-6 concentration in those with infection was 385 u/ml, compared with 314 u/ml in those with nonsignificant growth or no growth (*P *= 0.37) (Figure 6). Where repeat bronchoscopy was performed, none of the patients who grew pathogenic bacteria on the first lavage repeatedly grew the same isolate. The BALF KL-6 level was 298 u/ml in patients with direct lung injury and was 329 u/ml in those with indirect lung injury (*P *= 0.48) (data not shown).

## Discussion

In the present study we have extended previous observations about KL-6 in both the plasma and the BALF from patients with ARDS compared with normal individuals and at-risk individuals. Previous studies in adults with ALI used a bronchoscopic microsampling probe for estimating the epithelial lining oedema fluid KL-6 concentrations. Bronchoalveolar lavage represents the better validated technique and allows both sequential assessments and comparison with both normal individuals and at-risk individuals. In our patients the levels of KL-6 in the plasma reflected the severity of lung injury and were highest in those that subsequently died. The fact that KL-6 was not elevated in the plasma of an at-risk group of patients who were ventilated suggests that increased plasma levels of KL-6 reflect the pathophysiology of lung injury rather than representing a nonspecific effect of mechanical ventilation or critical illness.

There is considerable speculation about the mechanism whereby the KL-6 concentration is elevated in the plasma of patients with ARDS. Until recently the primary cellular source of KL-6 was thought to be type II pneumocytes [7] and the concentration of KL-6 was estimated to be extremely high in epithelial lining fluid [8,9]. It has been suggested that an increase in circulating KL-6 levels in interstitial pneumonitis is therefore due to an increase in KL-6 production by regenerating alveolar type II pneumocytes and/or to an enhanced permeability following destruction of the air–blood barrier in the affected lungs [15]. Recent data suggest that the KL-6 levels may indicate interstitial lung disease in patients since KL-6 is a potent proproliferative and antiapoptotic agent upon lung fibroblasts. Interestingly the effects of KL-6 were comparable in magnitude with those of transforming growth factor beta, basic fibroblast growth factor and platelet-derived growth factor, supporting the intriguing possibility that KL-6 may be a driver of fibroproliferation seen in ARDS patients. Whether KL-6 in the plasma predicts the development of persistent fibroproliferative ARDS is worthy of further study.

Plasma levels of KL-6 have been previously shown to correlate with indices of alveolar–capillary permeability, suggesting a link between serum KL-6 and alveolar epithelial barrier dysfunction. Circulating levels of KL-6 have been used as both a diagnostic tool and a prognostic tool in a variety of interstitial pneumonitis, sarcoidosis and alveolar proteinosis. Two previous studies of KL-6 in plasma of ARDS patients have been published. In the first, plasma levels of KL-6 correlated with the oxygenation index and were significantly elevated by day 4. In the more recent study in children with ARDS, there was a relationship between KL-6 and both the oxygenation index and survival. Neither of these studies looked contemporaneously at the alveolar compartment. A further study used bronchoscopic micro-sample probe to sample oedema fluid early in the course of ARDS [16]. In that study, plasma and epithelial lining fluid levels were elevated in nonsurvivors and alveolar epithelial type II cell production of KL-6 was shown to be cytokine responsive. For this reason we assessed whether there was a relationship between inflammation and lung levels of KL-6.

As might have been expected, the BALF levels of KL-6 were significantly higher than those of either normal individuals or at-risk control individuals. The BALF levels were significantly higher in ARDS patients than the contemporaneous plasma levels. Our data support the interpretation that the plasma KL-6 level reflects the lung level as they correlate together, although we did not demonstrate a significant transpulmonary gradient. Within the lung the BALF levels did not reflect the severity of lung injury or the presence of infection *per se*. The BALF levels did both reflect outcome and correlated with the degree of cellular inflammation/neutrophilic activation (myeloperoxidase) and chemokine levels (ENA-78). There was also a significant relationship between BALF VEGF and BALF KL-6. Since *in situ *hybridization studies have demonstrated that alveolar type II cells are the predominant source of VEGF within the lung, this correlation supports type II epithelial cells as the main (but not necessarily sole) cellular source of KL-6 within the lung in ARDS. Nevertheless since neutrophilic inflammation is an important determinant of alveolar capillary damage, the relationship with alveolar inflammation suggests that BALF KL-6 cannot simply be regarded as a marker of regenerating epithelial cells.

The present study has several limitations. Firstly, our sequential assessment of KL-6 by bronchoscopy was only possible in one-half of the patients due to early death, clinical improvement or contraindications to bronchoscopy. This level of dropout, however, is in keeping with other published bronchoscopic studies in ARDS [3,17]. Secondly, although the lung is considered the predominant source of KL-6, recent research has suggested other sites of production [18]. Therefore, although we found that plasma and BALF levels correlated with each other, we cannot be entirely sure of the cellular source of plasma KL-6. This uncertainty could potentially be important as it may limit the potential utility of plasma KL-6 as a marker of lung epithelial damage in ARDS.

## Conclusion

The present study demonstrated for the first time that the BALF KL-6 concentration is elevated in patients with ARDS but is not elevated in critically ill patients at risk of the disease. BALF KL-6 correlated with plasma KL-6 and was related to the severity of inflammation. Alveolar infection does not seem to determine the levels of KL-6 in either BALF or plasma. These observations support a role of proliferating, stimulated and or injured pulmonary epithelial cells in the pathogenesis of ALI. KL-6 may represent a useful marker of alveolar type II cell dysfunction in ARDS since levels reflect the severity of lung injury and neutrophilic inflammation. These data also suggest that KL-6 release across the alveolar epithelial barrier is associated with a poor prognosis. The pathophysiological roles of KL-6 in the development of ARDS warrant further study.

## Key messages

• KL-6 is elevated in the plasma and BALF of patients with ARDS.

• Plasma levels of KL-6 reflect the severity of lung injury and show that BALF levels correlate with indices of inflammation.

• BALF KL-6 levels of ARDS patients with significant pathogenic bacterial growth were similar compared with those without significant infection.

• KL-6 levels are higher in those who die than in those who survive.

• KL-6 release across the alveolar epithelial barrier is associated with a poor prognosis.

## Abbreviations

ALI = acute lung injury; ARDS = acute respiratory distress syndrome; BALF = bronchoalveolar lavage fluid; CI = confidence interval; ELISA = enzyme-linked immunosorbent assay; ENA-78 = epithelial-cell-derived neutrophil attractant 78; IL = interleukin; KL-6 = Kerbs von Lungren 6 antigen; VEGF = vascular endothelial growth factor.

## Competing interests

The authors declare that they have no competing interests.

## Authors' contributions

NN designed the study, recruited patients, performed the analyses, analysed the data and helped to write the manuscript. GDP helped to recruit patients, analysed the data and helped to write the data. WT, NM and MM identified and recruited patients, interpreted the data and helped to write the paper. DRT designed the study, analysed and interpreted the data and wrote the paper. DRT acts as guarantor for the study.
